# Colocalization coefficients evaluating the distribution of molecular targets in microscopy methods based on pointed patterns

**DOI:** 10.1007/s00418-016-1467-y

**Published:** 2016-07-26

**Authors:** Lukáš Pastorek, Margarita Sobol, Pavel Hozák

**Affiliations:** 1 Department of Biology of the Cell Nucleus, Institute of Molecular Genetics ASCR v.v.i., Vídeňská 1083, 142 20 Prague 4, Czech Republic; 2 Microscopy Centre, Institute of Molecular Genetics ASCR v.v.i., Vídeňská 1083, 142 20 Prague 4, Czech Republic; 3 Laboratory of Epigenetics of the Cell Nucleus, Division BIOCEV, Institute of Molecular Genetics of the ASCR v. v. i., Průmyslová 595, 252 50 Vestec, Czech Republic

**Keywords:** Colocalization, Quantitative analysis, Pointed patterns, Transmission electron microscopy, Manders’ coefficients, Immunohistochemistry

## Abstract

In biomedical studies, the colocalization is commonly understood as the overlap between distinctive labelings in images. This term is usually associated especially with quantitative evaluation of the immunostaining in fluorescence microscopy. On the other hand, the evaluation of the immunolabeling colocalization in the electron microscopy images is still under-investigated and biased by the subjective and non-quantitative interpretation of the image data. We introduce a novel computational technique for quantifying the level of colocalization in pointed patterns. Our approach follows the idea included in the widely used Manders’ colocalization coefficients in fluorescence microscopy and represents its counterpart for electron microscopy. In presented methodology, colocalization is understood as the product of the spatial interactions at the single-particle (single-molecule) level. Our approach extends the current significance testing in the immunoelectron microscopy images and establishes the descriptive colocalization coefficients. To demonstrate the performance of the proposed coefficients, we investigated the level of spatial interactions of phosphatidylinositol 4,5-bisphosphate with fibrillarin in nucleoli. We compared the electron microscopy colocalization coefficients with Manders’ colocalization coefficients for confocal microscopy and super-resolution structured illumination microscopy. The similar tendency of the values obtained using different colocalization approaches suggests the biological validity of the scientific conclusions. The presented methodology represents a good basis for further development of the quantitative analysis of immunoelectron microscopy data and can be used for studying molecular interactions at the ultrastructural level. Moreover, this methodology can be applied also to the other super-resolution microscopy techniques focused on characterization of discrete pointed structures.

## Introduction

In biomedical studies, the *colocalization* is commonly understood as the overlap between signals produced by distinctive dyes or stains in images. This term is currently associated especially with evaluating the immunostaining in images acquired using fluorescence microscopy (FM). There are several reviews describing the methods for quantitative interpretation of the fluorescent image data (Bolte and Cordelieres 2006; Comeau et al. 2006; Dunn et al. 2011; Scriven et al. 2008; Zinchuk et al. 2007). Nevertheless, the computational evaluation is inevitably accompanied by various sources of possible uncertainty and bias, such as bleeding-through of the fluorescence emission in different channels or out-of-focus signal. One must consider as well an impact of low resolution and image quality (lossy compression, noisy images, oversaturation) and an influence of “human factor” (subjective selection of thresholding, parameter settings, and colocalization method, Dunn et al. 2011). Furthermore, another issue represents the subjective or algorithmic selection of the region of interest (Jaskolski et al. 2005; Lachmanovich et al. 2003; Ramirez et al. 2010). Also, the merged fluorescent images can result in optical illusions, which in turn represent the misleading puzzle for the human visual perception and brain. All these factors may negatively bias the assessment of the colocalization and influence scientific conclusions.

Immunolabeling of tissues or cells in transmission electron microscopy (EM) is an alternative approach which allows to focus on subcellular compartments with much higher resolution. This approach shares many principal molecular mechanisms with the immunolabeling technique in the light microscopy. The molecules of interest, called antigens, are recognized and marked by specific antibodies. Consequently, the subcellular location of the antigen can be detected. However, the method of detection is different in FM and EM. The visualization technique in EM requires the presence of an electron-dense probe which increases electron scatter resulting in dark spots of the high contrast. In such a way, the target molecules become visible due to the colloidal metal particles which are conjugated with the antibodies. The different types of particles of the various size, shape, and material can substitute the different colors of fluorochromes used for labeling in light microscopy (Philimonenko et al. 2014).

Various methods of the correlative microscopy experiments are currently in use with the aim to describe the biological context at various scales using combination of different microscopy techniques (Caplan et al. 2011; Plitzko et al. 2009; Sartori et al. 2007). However, the EM approach is still characterized by a higher resolution and more precise detection of molecules as compared to FM technique, super-resolution microscopy included. Nevertheless, the evaluation of the immunolabeling patterns in EM is still under-investigated and biased by the subjective judgement and non-quantitative interpretation of the image data. On the other hand, there are several quantitative approaches in EM focused on the spatial point pattern analysis and testing the statistical significance in the spatial distribution (Anderson et al. 2003; Glasbey and Roberts 1997; Hoskins et al. 2013; Mayhew 2011a, b, 2015; Philimonenko et al. 2000; Schöfer et al. 2004). The most known techniques are based on the quadrat count analysis and the multi-distance neighbor principle. Both approaches comprise the same conceptual structure: the comparison of the empirical frequency distributions of the detected particles with the theoretical situations, where no clustering or colocalization is assumed. The term “clustering” is used for patterns generated by the physiological processes occurring in “non-random” way, and the elements of investigated pattern are directly or indirectly dependent to other elements of the same type in the process. From the statistical view, the spatial clustering is not directed by the random homogenous Poisson process. For the conceptual clarity, we will refer to Poisson process as “random” and to clustering as “non-random” process. Poisson point process always results in the complete randomness and stochastically independence between the locations of the points. This independence means that the occurrence of one particle or event does not influence the occurrence of the other one. In spatial statistics, we conceptually test and calculate the level of the agreement between the investigated experimental process and the theoretical random process. In the first approach—the quadrat analysis—the chosen grid structure is projected onto the image (the image is notionally cut into squares of the equal size) and the frequencies of the detected particles in the individual square regions on a grid are detected and compared with the predicted mean frequency representing the result of theoretical random process. The second approach is based on the comparison of the empirical frequencies of the pairs of particles on the chosen distance with the theoretical frequency distribution of the pairs (the null hypothesis of the complete spatial randomness in the distribution of the particles). These methods (reviewed in (D’Amico and Skarmoutsou 2008a, b) are focused on the evaluation of the statistical significance of the spatial patterns. However, these approaches were not originally designed to provide the information about the level of the association between different types of particles. That is why we propose a unifying methodology including the definitional scheme and colocalization coefficients, which extends existing significance testing approaches. The proposed methodology follows the idea incorporated in the widely used FM Manders’ colocalization coefficients (MCC, (Manders et al. 1993) and establishes its statistical counterpart for EM and other microscopy methods based on pointed pattern.

## Materials and methods

### Theoretical background

Our approach is based on the redefinition of the term *colocalization* understood mostly as the spatial overlap of the labels from different color channels in identical pixel positions in an image. The suggested definition emphasizes more physical and probability-based *systematic spatial co-distribution (co-occurrence)* of the molecules of the interest, which are labeled by the metal particles of different types. This stochastic *co-occurrence* emerges from the individual direct or indirect molecular interactions.

 We propose a methodology which stresses the colocalization (systematic spatial co-distribution) as the product of the spatial interactions at the *single*-*molecule level*. The global spatial co-organization results from the local co-presence between smaller components (molecules). The described approach combines the idea of the global colocalization between labelings in a cell or subcellular compartment with the idea of the local molecular interactions—*single*-*molecule colocalization*. Since we can detect an antigen only by a detection system which incorporates the metal nanoparticles, we prefer instead the term *single*-*particle colocalization*.

Definition of the single-particle colocalization: *A chosen single particle of type A colocalizes with the given single particle of type B only if the distance between them is inside the defined distance range.We denote each of those two particles as “colocalizing particles,” which form together “colocalizing pair.”*

The particles are not assumed in the calculations as the physical objects, but as centroids—the points in the space associated with a given particle through the index number (identifier). The expression *aggregated colocalization* is used in this study as the quantified average ratio between the different types of labels. We conclude that the *aggregated colocalization* is the consequence of the collective behavior of the *local paircolocalizations* between the single particles of different types.

The concept uses the input data on the relevant distance intervals of the interest from the approach described in Philimonenko et al. (2000), where the normalized pair-correlation and pair cross-correlation functions are calculated, visualized, and statistically tested using the Monte Carlo estimates of two-sided 95 % confidence intervals. Therefore, the proposed coefficients require the distance interval for single-particle colocalization to be chosen as the input parameter for analysis.

### Computational description

To obtain a more complete overview of the global colocalization pattern resulting from the single-particle colocalizations, we divide the quantitative analysis of the image data into three stages.

#### Frequency distribution of particles

At the first stage, we characterize the average absolute number, ${\text{CC}}_{1}^{{{\text{abs}}\left( {A + B} \right)}}$, and average proportion of the particles of type *A* and type *B*, ${\text{CC}}_{1}^{{{\text{rel}}\left( A \right)}}$ and ${\text{CC}}_{1}^{{{\text{rel}}\left( B \right)}}$, per image.

1 $${\text{CC}}_{1}^{{{\text{abs}}\left( {A + B} \right)}} = \frac{1}{k}\sum\limits_{i = 1}^{k} {\left( {n_{i}^{A} + n_{i}^{B} } \right)} ,$$

2 $${\text{CC}}_{1}^{{{\text{rel}}\left( A \right)}} = \frac{1}{k}\sum\limits_{i = 1}^{k} {\frac{{n_{i}^{A} }}{{n_{i}^{A} + n_{i}^{B} }},}$$

3 $${\text{CC}}_{1}^{{{\text{rel}}\left( B \right)}} = \frac{1}{k}\sum\limits_{i = 1}^{k} {\frac{{n_{i}^{B} }}{{n_{i}^{A} + n_{i}^{B} }}} ,$$

where $k$ is the number of evaluated images and $i$ is the index of the image (identifier), in which $i = 1,2, \ldots ,k$. The symbols $n_{i}^{A}$ and $n_{i}^{B}$ are the absolute frequencies of the particles of type *A* and type *B* on the $i$th image. Calculation of these simple coefficients is essential for the interpretation of the coefficients at following stages.

#### Relative colocalization coefficient (colocalization ratio)

At the second stage, we examine the average fraction of the colocalizing particles of type *A* and the average fraction of the colocalizing particles of type *B* per image (*relative aggregated colocalization*). The coefficients at this stage are denoted as ${\text{CC}}_{2}$. The *relative aggregated colocalization* is the percentage of the particles of type *A* or type *Bcolocalizing at least with one* particle of the other type. The *absolute aggregated colocalization* is expressed as the absolute number of the colocalizing particles of the given type.

On the one hand, the information about the *average fraction* of the particles of type *B* colocalizing with the *average fraction* of the particles of type *A* per single image is descriptively reversible in interpretation. On the other hand, we should be cautious about this interpretation, as the semantic coupling of the aggregated average ratios can induce the incorrect dependence and mask the possible statistical relationships among average ratios in different images.

Also, the colocalization of particles in the colocalizing pair is symmetric (each particle of the pair is colocalizing particle). However, the relation is not symmetric, if we are interested in the colocalization of the single particle of type *A* of the chosen colocalizing pair and all other particles of type *B* and vice versa. This asymmetry implies the principal difference in the meaning of the coefficients ${\text{CC}}_{2}^{A}$ and ${\text{CC}}_{2}^{B}$ proposed at this stage. When compared with the simple relative coefficients ${\text{CC}}_{1}$, the values of the ${\text{CC}}_{2}$ are not complementary to each other (the sum of ${\text{CC}}_{2}^{A}$ and ${\text{CC}}_{2}^{B}$ is not equal to one).

To avoid this interpretative pitfall and highlight the difference between the coefficients describing the *relative aggregated colocalizations*, we prefer to establish the electron microscopy colocalization coefficient of the particles of type *Baround* the particles of type *A* as ${\text{CC}}_{2}^{A}$ and of the particles of type *Aaround* the particles of type *B* as ${\text{CC}}_{2}^{B}$.

The coefficients are defined in this manner as

4 $${\text{CC}}_{2}^{A} = \frac{1}{k}\sum\limits_{i = 1}^{k} {\sum\limits_{j} {\frac{{f\left( {{\mathbf{x}}_{ij}^{A} } \right)}}{{n_{i}^{A} }},} }$$

where

5 $$\forall f\left( {{\mathbf{x}}_{ij}^{A} } \right):f\left( {{\mathbf{x}}_{ij}^{A} } \right) = \left\{ {\begin{array}{*{20}l} 1 \hfill & {\left[ {\sum\limits_{q} {I\left( {r^{\prime} \le d\left( {{\mathbf{x}}_{ij}^{A} ,{\mathbf{x}}_{iq}^{B} } \right) < r^{\prime\prime}} \right)} } \right] > 0} \hfill \\ 0 \hfill & {\text{otherwise}} \hfill \\ \end{array} } \right.,$$

where symbols $k$, $i$, $n_{i}^{A}$ have the same meaning as in the first stage coefficients, $j$ is the index (identifier) of the particle on the $i$th image, in which $j = 1,2, \ldots ,n_{i}^{A}$, and $q$ is the index of the particle of type *B* on the $i$th image, in which $q = 1,2, \ldots ,n_{i}^{B}$. The expression $d( {{\mathbf{x}}_{ij}^{A} ,{\mathbf{x}}_{iq}^{B} } )$ is the Euclidean (straight-line) distance between the $j$th particle of type *A* on the $i$th image (coordinates encoded in the vector ${\mathbf{x}}_{ij}^{A}$) and $q$th particle of type *B* (coordinates encoded in vector ${\mathbf{x}}_{iq}^{B}$) on the $i$th image; $r^{\prime}$ and $r^{{\prime \prime }}$ are the chosen distance limits (distance range). If the Euclidean distance between vectors ${\mathbf{x}}_{ij}^{A}$ and ${\mathbf{x}}_{iq}^{B}$ meets the conditions inside the parentheses $( {r^{{\prime }} \le d( {{\mathbf{x}}_{ij}^{A} ,{\mathbf{x}}_{iq}^{B} } ) < r^{{\prime \prime }} } )$ and is inside the chosen range, the step function $I( {r^{{\prime }} \le d( {{\mathbf{x}}_{ij}^{A} ,{\mathbf{x}}_{iq}^{B} } ) < r^{{\prime \prime }} } )$ is equal to one (given pair of particles is *colocalizing pair*). Otherwise, the step function $I$ is equal to zero (given pair of particles *does notcolocalize*). The symbol $f( {{\mathbf{x}}_{ij}^{A} } )$ defines the second step function, which evaluates the number of particles of type *B* colocalizing with particle ${\mathbf{x}}_{ij}^{A}$. If the particle of type *A* colocalizes at least with one particle ${\mathbf{x}}_{iq}^{B}$, the step function $f( {{\mathbf{x}}_{ij}^{A} } )$ is equal to one. Otherwise, the particle of type *A* is linked with the value of step function equal to zero (given particle of type *A* does not colocalize with any particle of type *B*).

##### The pseudocode for coefficient ${\text{CC}}_{2}^{A}$

The formula ${\text{CC}}_{2}^{A}$ can be described also by the structured algorithmic steps of the operating computational principle. The visualization of the described pseudocode for the readers with purely biological/biomedical background is illustrated in Fig. 2. The formula consists of the following steps:

1. Select image with index (identifier) $i$.
2. Choose particle of type *A* with index $j$ with coordinates ${\mathbf{x}}_{ij}^{A}$ on the selected $i$th image.
3. Select particle of type *B* with index $q$ with coordinates ${\mathbf{x}}_{iq}^{B}$ from the $i$th image and calculate distance between this particle and particle chosen in Step 2: $d( {{\mathbf{x}}_{ij}^{A} ,{\mathbf{x}}_{iq}^{B} })$.
4. If their distance is inside the user’s defined distance interval, pair of the particles is *colocalizing pair* and function $I( {r^{\prime} \le d( {{\mathbf{x}}_{ij}^{A} ,{\mathbf{x}}_{iq}^{B} } ) < r^{\prime\prime}}) = 1.$ If it is non-colocalizing pair (distance is less or more than limits), function is equal to zero.
5. Repeat Steps 3–4 for each particle of type *B* on the chosen $i$th image: $q = 1,2, \ldots ,n_{i}^{B}$ (evaluate which particles of type *B* are *colocalizing* particles with particle of type *A* chosen in Step 2).
6. Compute the number (sum) of colocalizing particles of type *B* on the selected $i$th image, which colocalize with particle chosen in Step 2: $\sum\limits_{q} {I( {r^{\prime} \le d( {{\mathbf{x}}_{ij}^{A} ,{\mathbf{x}}_{iq}^{B} }) < r^{\prime\prime}} )}$.
7. If value of function calculated in Step 6 is nonzero, function $f( {{\mathbf{x}}_{ij}^{A} } ) = 1$. In other case, $j$th particle colocalizes with NO particles and function $f( {{\mathbf{x}}_{ij}^{A} }) = 0$.
8. Repeat Steps 2–7 for all particles of type *A* on the $i$th selected image: $j = 1,2, \ldots ,n_{i}^{A}$.
9. Calculate the number (sum) of the particles of type *A* on the given image, which colocalize at least with one particle of type *B*: $\sum\limits_{j} {f\left( {{\mathbf{x}}_{ij}^{A} } \right)}$. These particles of type *A* were associated with nonzero values of function $f\left( {{\mathbf{x}}_{ij}^{A} } \right)$.
10. Consequently, this sum is expressed as a proportion of the number of all particles of type *A* on the given image: $\sum\limits_{j} {\frac{{f( {{\mathbf{x}}_{ij}^{A} } )}}{{n_{i}^{A} }}}$.
11. Repeat Steps 1–10 for all analyzed images: $i = 1,2, \ldots ,k$.
12. After iterative calculation of ratio from Step 10 for each image, the total average ratio (total proportion) is computed (dividing the total sum by the number of analyzed images $k$).

For the mathematical definition of the coefficient ${\text{CC}}_{2}^{B}$, we interchange semantically and symbolically the superscripts *B* and *A* in all formulas and steps.

##### Summary colocalization coefficients and derived properties

**Fig. 1:**
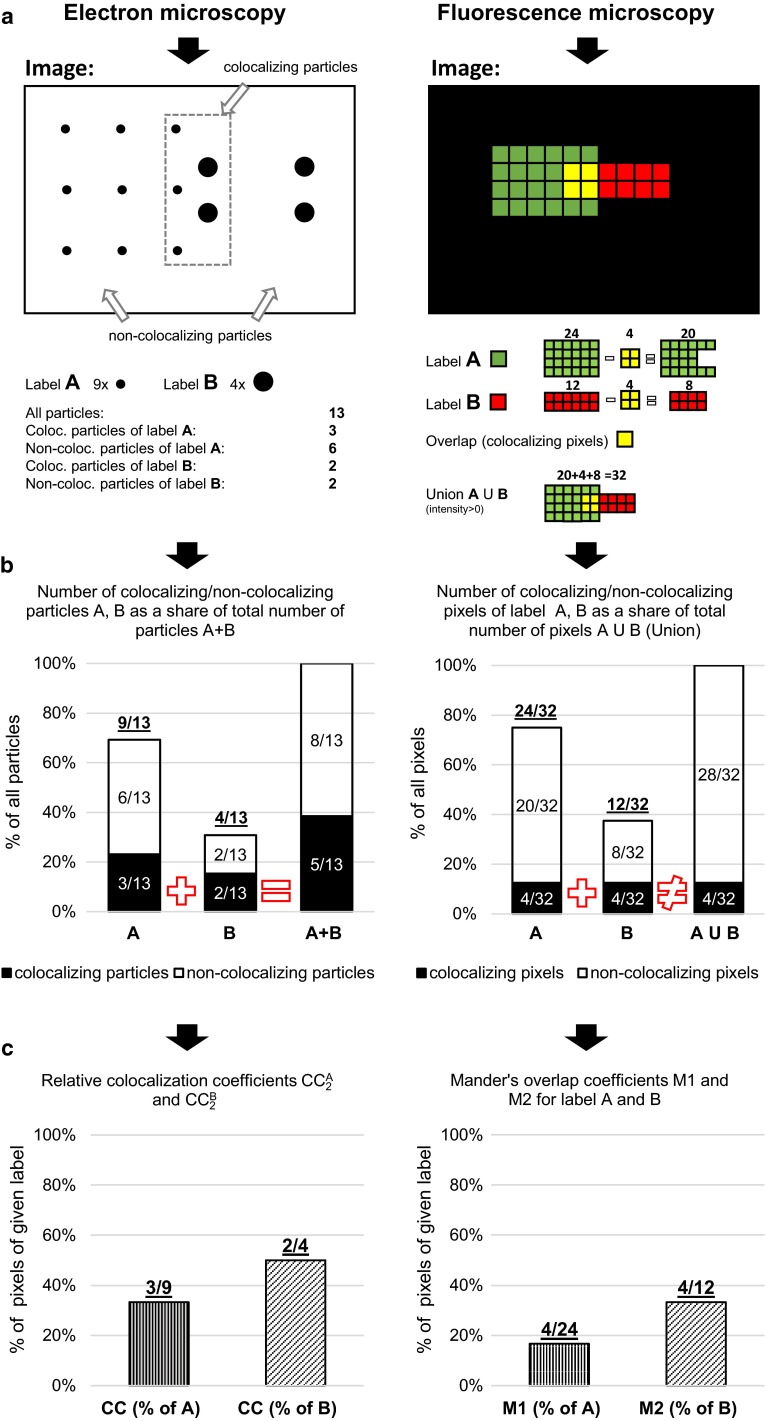
Conceptual comparison of EM colocalization coefficients versus the overlap in FM. **a** The colocalization in EM is based on the single-particle colocalization resulting in colocalizing pairs of particles on the given distance range. On the contrary, FM colocalization is based on the overlapping signals in pixels from the different color channels. **b** The relative frequency distribution of the colocalizing/non-colocalizing particles/pixels of the model examples in (*A*). After combining the information about the colocalizing particles, we conclude the additive characteristics of EM colocalization versus nonadditive but union characteristics of FM colocalization. Additionally, the first two bars for labels *A* and *B* in the *bar graph* from EM image include the relations between proposed coefficients: ${\text{CC}}_{A/(A + B)}^{\text{coloc}} + {\text{CC}}_{A/(A + B)}^{\text{non-coloc}} = {\text{CC}}_{1}^{{{\text{rel}}\left( A \right)}}$ and ${\text{CC}}_{B/(A + B)}^{\text{coloc}} + {\text{CC}}_{B/(A + B)}^{\text{non-coloc}} = {\text{CC}}_{1}^{{{\text{rel}}\left( B \right)}}$ (Eqs. 11, 12). **c** The Manders’ overlap coefficients in FM are calculated based on the intersection of the signals (overlap) and the union of the overlapped plus non-overlapped pixels of the labels in (*A*). The Manders’ overlap coefficient M1 for the label *A* can be alternatively calculated as division of the proportions (4/32)/(24/32) = 4/24, that is, 12.5 %/75.0 % ≈ 16.7 %. Coefficient M2 for the label B is: (4/32)/(12/32) = 4/12, that is, 12.5 %/37.5 % ≈ 33.3 %. The EM relative aggregated colocalization coefficients ${\text{CC}}_{2}^{A}$ for the label *A* and ${\text{CC}}_{2}^{B}$ for the label *B* are calculated in the similar manner from the frequency distribution of the colocalizing and non-colocalizing particles of the corresponding type (Eq. 4). Also, the alternative calculation is based on the identical approach as in FM. On the other hand, we can characterize more precisely this ratio using our proposed definitions: ${\text{CC}}_{A/(A + B)}^{\text{coloc}} /{\text{CC}}_{1}^{{{\text{rel}}\left( A \right)}}$ and ${\text{CC}}_{B/(A + B)}^{\text{coloc}} /{\text{CC}}_{1}^{{{\text{rel}}\left( B \right)}}$ (Eqs. 13, 14) based on the data from (A) and (B)

To describe the colocalization situation more detailed, we derive another important statistical coefficients, which includes the integration of the first and second level coefficients. By this combination, we receive the set of four summary measures, which includes the complex information about the proportions of *colocalizing* particles (${\text{CC}}_{A/(A + B)}^{\text{coloc}}$, ${\text{CC}}_{B/(A + B)}^{\text{coloc}}$) and *non*-*colocalizing* particles (${\text{CC}}_{A/(A + B)}^{\text{non-coloc}}$, ${\text{CC}}_{B/(A + B)}^{\text{non-coloc}}$) of the given labeling on the average image (Fig. 1):

6 $${\text{CC}}_{A/(A + B)}^{\text{coloc}} = {\text{CC}}_{1}^{{{\text{rel}}\left( A \right)}} \cdot {\text{CC}}_{2}^{A} ,$$

7 $${\text{CC}}_{A/(A + B)}^{\text{non-coloc}} = {\text{CC}}_{1}^{{{\text{rel}}\left( A \right)}} \cdot \left( {1 - {\text{CC}}_{2}^{A} } \right),$$

8 $${\text{CC}}_{B/(A + B)}^{\text{coloc}} = {\text{CC}}_{1}^{{{\text{rel}}\left( B \right)}} \cdot {\text{CC}}_{2}^{B} ,$$

9 $${\text{CC}}_{B/(A + B)}^{\text{non-coloc}} = {\text{CC}}_{1}^{{{\text{rel}}\left( B \right)}} \cdot \left( {1 - {\text{CC}}_{2}^{B} } \right).$$

From the set of equations, we can conclude following intrinsic properties:

10 $${\text{CC}}_{A/(A + B)}^{\text{coloc}} + {\text{CC}}_{A/(A + B)}^{\text{non-coloc}} + {\text{CC}}_{B/(A + B)}^{\text{coloc}} + {\text{CC}}_{B/(A + B)}^{\text{non-coloc}} = 1,$$

11 $${\text{CC}}_{1}^{{{\text{rel}}\left( A \right)}} = {\text{CC}}_{A/(A + B)}^{\text{coloc}} + {\text{CC}}_{A/(A + B)}^{\text{non-coloc}} ,$$

12 $${\text{CC}}_{1}^{{{\text{rel}}\left( B \right)}} = {\text{CC}}_{B/(A + B)}^{\text{coloc}} + {\text{CC}}_{B/(A + B)}^{\text{non-coloc}} ,$$

13 $${\text{CC}}_{2}^{A} = \frac{{{\text{CC}}_{A/(A + B)}^{\text{coloc}} }}{{{\text{CC}}_{1}^{{{\text{rel}}\left( A \right)}} }},$$

14 $${\text{CC}}_{2}^{B} = \frac{{{\text{CC}}_{B/(A + B)}^{\text{coloc}} }}{{{\text{CC}}_{1}^{{{\text{rel}}\left( B \right)}} }}.$$

The defined coefficients are complementary to ${\text{CC}}_{1}^{{{\text{abs}}\left( {A + B} \right)}}$ and avoid the user’s incorrect explanation of the importance of the recognized colocalization.

##### Relative density of colocalization

**Fig. 2:**
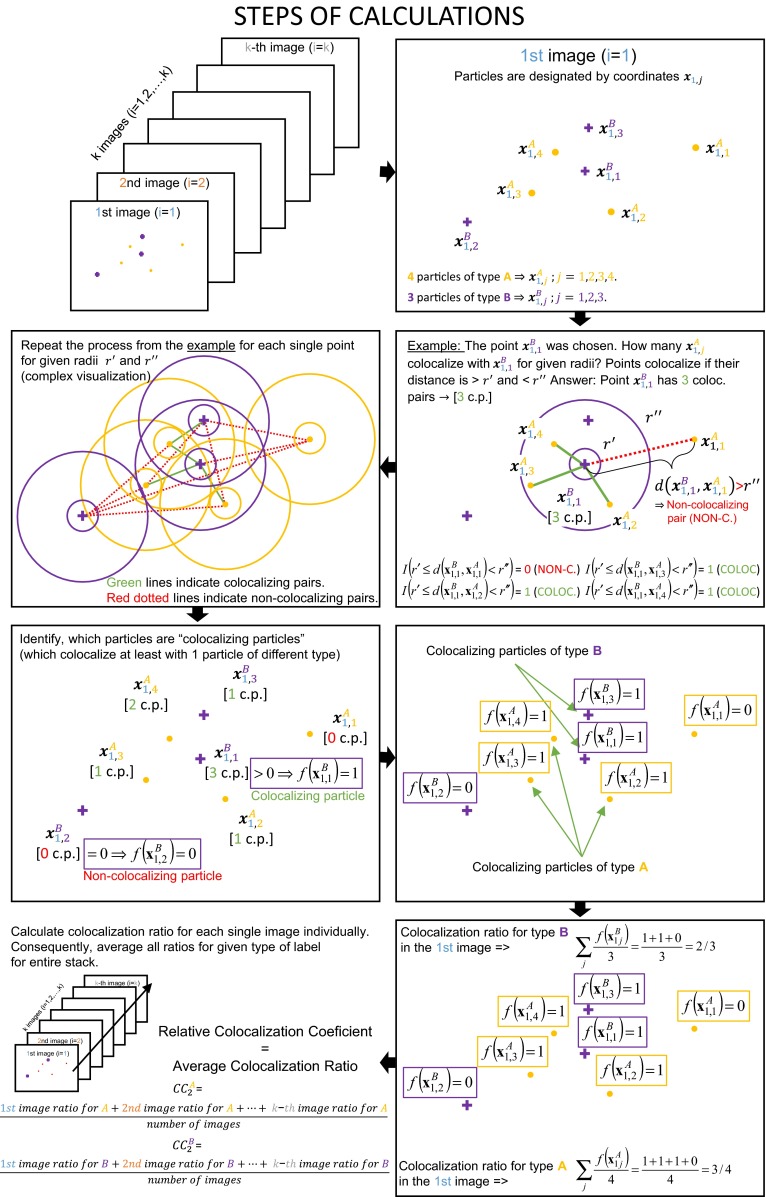
Example of the calculations of the proposed EM coefficients on the model image. The particles of the labels *A* and *B* are represented by their coordinates ${\mathbf{x}}_{ij}^{A}$ and ${\mathbf{x}}_{ij}^{B}$ as purple crosses and yellow dots. We consider only the first image as example, so the parameter $i = 1$. The parameter $j$ is the identifier of the particle (${\mathbf{x}}_{ij}^{A}$ is the $j$th particle of type *A* on the $i$th image, e.g., ${\mathbf{x}}_{1,2}^{A}$ is the second particle of type *A* on the first image). The number of the particles of the label A is four ($n_{1}^{A} = 4$), and there are three particles of the label B ($n_{1}^{B} = 3$). An image includes four colocalizing pairs of the labels A and B, where three colocalizing particles are of type *A* (${\mathbf{x}}_{1,2}^{A} ;{\mathbf{x}}_{1,3}^{A} ;{\mathbf{x}}_{1,4}^{A}$) and two colocalizing particles are of type *B* (${\mathbf{x}}_{1,1}^{B} ;{\mathbf{x}}_{1,3}^{B}$). If we assume the toy example that the first image is also the only image of the stack $\left( {k = 1} \right)$, we can calculate all coefficients for this single image. Because of our toy example, the last averaging operation illustrated in the figure is omitted (denominator would be equal to number one). Coefficient ${\text{CC}}_{1}^{{{\text{abs}}\left( {A + B} \right)}} = 7$ (absolute number of particles, Eq. 1); ${\text{CC}}_{1}^{{{\text{rel}}\left( A \right)}} = 4/7$; ${\text{CC}}_{1}^{{{\text{rel}}\left( B \right)}} = 3/7$ (average proportion of the particles, Eq. 2, 3); ${\text{CC}}_{2}^{A} = 3/4$; ${\text{CC}}_{2}^{B} = 2/3$ (relative colocalization fractions, Eq. 4); ${\text{CC}}_{A/(A + B)}^{\text{coloc}} = (3/4)*(4/7)$; ${\text{CC}}_{B/(A + B)}^{\text{coloc}} = (2/3)*(3/7)$ (summary coefficients describing the colocalizing fractions in their relation to the total number of particles on image, Eq. 6, 8) ${\text{CC}}_{A/(A + B)}^{\text{non-coloc}} = (3/4)*\left[ {1 - (4/7)} \right]$; ${\text{CC}}_{B/(A + B)}^{\text{non-coloc}} = (2/3)*\left[ {1 - (3/7)} \right]$ (non-colocalizing fractions as part of total number of particles on image, Eqs. 7, 9); ${\text{CC}}_{3}^{B/A} = (2 + 1 + 1)/(3*3)$; ${\text{CC}}_{3}^{A/B} = (3 + 1)/(2*4)$ (relative densities of colocalization, Eqs. 15, 16)

At the final third stage, we study the *average relative densityof thecolocalization,* designated as ${\text{CC}}_{3}^{B/A}$ and ${\text{CC}}_{3}^{A/B}$. The coefficients describe the average fraction of particles of type *B* (type *A*) colocalizing around the average single particle of type *A* (type *B*). The coefficients are calculated first for an image and then for a set of images. In other words, the coefficients are used to quantify and assess the level of spatial co-distribution at the single-molecule level (average spatial density) through the whole set of image data. The relation between the coefficients is asymmetric and not complementary. The coefficients are defined as

15 $${\text{CC}}_{3}^{B/A} = \frac{1}{k}\sum\limits_{i = 1}^{k} {\sum\limits_{j} {\left\{ {\frac{1}{{w({\mathbf{x}}_{ij}^{A} )}} \cdot \frac{{\left[ {\sum\limits_{q} {I\left( {r^{{\prime }} \le d\left( {{\mathbf{x}}_{ij}^{A} ,{\mathbf{x}}_{iq}^{B} } \right) < r^{{\prime \prime }} } \right)} } \right]}}{{n_{i}^{{{\text{col}}A}} \cdot n_{i}^{B} }}} \right\},} }$$

16 $${\text{CC}}_{3}^{A/B} = \frac{1}{k}\sum\limits_{i = 1}^{k} {\sum\limits_{j} {\left\{ {\frac{1}{{w({\mathbf{x}}_{ij}^{B} )}} \cdot \frac{{\left[ {\sum\limits_{q} {I\left( {r^{{\prime }} \le d\left( {{\mathbf{x}}_{ij}^{B} ,{\mathbf{x}}_{iq}^{A} } \right) < r^{{\prime \prime }} } \right)} } \right]}}{{n_{i}^{{{\text{col}}B}} \cdot n_{i}^{A} }}} \right\},} }$$

where $n_{i}^{{{\text{col}}A}}$ is the number of the particles of type *A* on the $i$th image, which colocalize at least with one particle of type *B* on the $i$th image and $n_{i}^{{{\text{col}}B}}$ is the number of the particles of type *B* on the $i$th image, which colocalize at least with one particle of type *A* on the $i$th image. The expressions $w({\mathbf{x}}_{ij}^{A} )$, $w({\mathbf{x}}_{ij}^{B} )$ denote values of any weight correction function, which corrects the negative image boundary effect for the particles near edges of image. The other variables are the same as described before. The coefficient ${\text{CC}}_{3}^{B/A}$ calculates the *average relative densityof thecolocalization* of the particles *B* around single particle *A*, and ${\text{CC}}_{3}^{A/B}$ enumerates the *average relative densityof thecolocalization* of the particles *A* around single particle *B*. The illustrative description of the principle of this calculation is shown in Fig. 1. The simplified model example and individual steps of this calculation are presented in Fig. 2.

### Description of the datasets

#### EM image data

The image dataset obtained using EM contained 21 images (1376 × 1032 pixels) with the randomly chosen rectangular segments of nucleoli. On the level of the entire dataset, 565 particles were detected and analyzed (53 % particles correspond to fibrillarin labeling and 47 % particles correspond to PIP2). Each image included 26.95 ± 13.64 particles, from this 14.33 ± 7.17 particles of fibrillarin and 12.67 ± 8.64 particles of PIP2 (arithmetic mean ± standard deviation). EM data were evaluated using all coefficients proposed in this paper and algorithmically implemented as the ImageJ plug-in. The significance of the colocalization on the various distance ranges was evaluated using pair-correlation function (Philimonenko et al. 2000).

#### FM image data

The FM image data were obtained using confocal microscopy (CM) and the super-resolution structured illumination microscopy (SIM). The analysis included the detection of the complete ovaloid nucleolar regions of interest (ROI) based on the nucleolar marker fibrillarin. Then, we processed the subcellular pixel intensity values of fibrillarin and PIP2 channels. We chose nine nucleolar regions for each imaging technique, covering 1.19 ± 1.13 % of the area of a confocal image and 1.22 ± 0.49 % of the area of a SIM image. SIM and confocal FM data were analyzed using the Manders’ overlap coefficients calculated directly based on the logical operations applied to the individual color channels. For less subjective and more representative evaluation, thresholded and also non-thresholded images were analyzed. Before calculation of the Manders’ overlaps, we applied automatic and user’s bias-free Costes thresholding (Costes et al. 2004) implemented in the software ImageJ 1.49q (plug-in JACoP, Bolte and Cordelieres 2006). This plug-in produces two possible outputs of thresholds for each pair of channels as a consequence of the ordering of the channel images in plug-in’s threshold analysis. We applied Costes thresholding based on the entire image data and also on the image data of the nucleolar regions of interest only. Accordingly, we obtained five sets of colocalization ratios: (1) for the non-thresholded image, (2) and (3) for two thresholded images based on the entire image data with Costes algorithm, and (4) and (5) for two thresholded images based on the image data of the regions of interest (nucleoli).

### Microscopy

#### Cell cultures

Human cervical carcinoma (HeLa, ATCC No. CCL2) cells and osteosarcoma (U2OS, ATCC No. HTB96) cells were grown in monolayer in D-MEM with 10 % fetal bovine serum (FBS) at 37 °C in 5 % CO_2_ humidified atmosphere. HeLa suspension cell culture was grown in S-MEM with 5 % FBS under the same conditions.

#### Confocal microscopy

U2OS cells were fixed with 4 % formaldehyde in PBS. Cells were then simultaneously permeabilized and blocked with 0.3 % Triton X-100 plus 5 % normal donkey serum (NDS) in PBS for 75 min. To ensure the blocking of unspecific interactions and sufficient antibody penetration, cells were incubated with primary antibodies diluted in 1 % bovine serum albumin (BSA) plus 0.3 % Triton X-100 in PBS overnight at +4 °C. After washing, cells were incubated with secondary antibodies diluted in 1 % bovine serum albumin (BSA) plus 0.3 % Triton X-100 in PBS for 1 h. Further cells were washed and mounted in Mowiol with 0.08 µg/ml DAPI. Images were acquired using confocal microscope TCS SP5 AOBS TANDEM with 100x (NA 1.4) oil immersion objective lens (Leica Microsystems GmbH, Wetzlar, Germany).

#### Super-resolution structured illumination microscopy

U2OS cells grown on high-performance cover glasses 18 × 18 mm^2^ with restricted thickness-related tolerance D = 0.17 mm ± 0.005 mm and refractive index = 1.5255 ± 0.0015 were processed similar to the samples for CM. Extensive washes were done in between all steps. Images were acquired using a super-resolution structured illumination microscope (ELYRA PS.1, Carl Zeiss; Andor iXon3 885 EMCCD camera, pixel size 8 × 8 μm) with Plan-Apochromat 63 ×/1.4 Oil DIC M27 oil immersion objective lens using the parameters as follows: number of SIM rotations = 5; SIM grating periods varied according to the excitation wavelength from 34.0 to 42.0 μm.

#### Transmission electron microscopy

HeLa cells were fixed in 3 % formaldehyde plus 0.1 % glutaraldehyde and embedded into LR White resin by a standard procedure (Sobol et al. 2010). For EM, HeLa cells were high-pressure frozen, freeze-substituted, and embedded into LR White resin according to a previously published protocol (Sobol et al. 2011). Ultrathin sections of 70 nm were examined in Morgagni 268 transmission electron microscope at 80 kV and Tecnai G2 20 LaB6 electron microscope at 200 kV (FEI, Eindhoven, The Netherlands). The images were captured with Mega View III CCD camera (pixel size 6.45 × 6.45 μm) and Gatan Model 894 UltraScan 1000 camera (pixel size 14 × 14 μm). Multiple sections of at least three independent immunogold labeling experiments were analyzed. Adobe Photoshop CS3 version 10.0 was used to highlight and magnify 6-nm gold particles in images with dots of the similar diameter as the 12-nm particles, but red color to facilitate their visualization in the images.

#### Antibodies

Primary antibodies are as follows: anti-PIP2 mouse monoclonal IgM antibody (ab11039, Abcam; 4 μg/ml for SIM, 16 μg/ml for CM, 32 μg/ml for EM), anti-fibrillarin rabbit monoclonal IgG antibody (2639, Cell Signaling Technology Inc.; 0.15 μg/ml for CM and SIM, 0.3 μg/ml for EM).

Secondary antibodies are as follows: donkey anti-mouse IgM conjugated with Cy3 (715-165-140, Jackson ImmunoResearch; 10 μg/ml for CM), goat anti-mouse IgM conjugated with Alexa 555 (A21426, Jackson, 10 μg/ml for SIM), donkey anti-rabbit IgG (H + L) conjugated with Alexa 488 (A-21206, Thermo Fisher Scientific; 5 μg/ml for CM), goat anti-rabbit IgG (H + L) conjugated with Alexa 647 (A-21245, Thermo Fisher Scientific; 5 μg/ml for SIM), goat anti-mouse IgM (μ-chain specific) antibody coupled with 12-nm (115-205-075) colloidal gold particles, goat anti-rabbit IgG (H + L) antibody coupled with 6-nm (111-195-144) colloidal gold particles (all gold-conjugated secondary antibodies were from Jackson ImmunoResearch Laboratories and diluted 1:30).

## Results and discussion

### Analyzed experimental data

To demonstrate the performance of the proposed coefficients for EM, we investigated the level of the spatial interaction of phosphatidylinositol 4,5-bisphosphate (PIP2) with fibrillarin in nucleoli and conceptually compared the results with the Manders’ colocalization coefficients and distributional data for fluorescent imaging.

Fibrillarin is an abundant nucleolar protein involved in site-specific 2′-*O*-ribose methylation and processing of pre-ribosomal RNA (pre-rRNA), which takes place in box C/D small nucleolar RNPs (Fatica et al. 2000; Rakitina et al. 2011). In interphase cells, fibrillarin localizes at the boundary between fibrillar centers (FCs) and the dense fibrillar component (DFC), which is a site of ribosomal DNA (rDNA) transcription, and in the DFC itself, where pre-rRNA processing occurs (Boisvert et al. 2007; Hernandez-Verdun 2006, 2011; Hozák et al. 1994; Ochs et al. 1985; Sobol et al. 2013).

Recently, we showed that PIP2 is involved in rDNA transcription and nucleolar organization throughout the cell cycle (Sobol et al. 2013; Yildirim et al. 2013). We revealed that PIP2 interacts with RNA polymerase I (Pol I) and upstream binding factor (UBF), involved in rDNA transcription, as well as with fibrillarin. We demonstrated that direct binding of PIP2 to UBF and fibrillarin changes their conformation affecting the binding to nucleic acids. Furthermore, we have shown that PIP2 associates with Pol I and UBF in a transcription-independent manner. On the other hand, association of PIP2 with fibrillarin is dependent on the production of rRNA (Sobol et al. 2013; Yildirim et al. 2013). In agreement with its functions, PIP2 localizes in FCs as well as in the DFC (Osborne et al. 2001; Sobol et al. 2013; Yildirim et al. 2013).

**Fig. 3:**
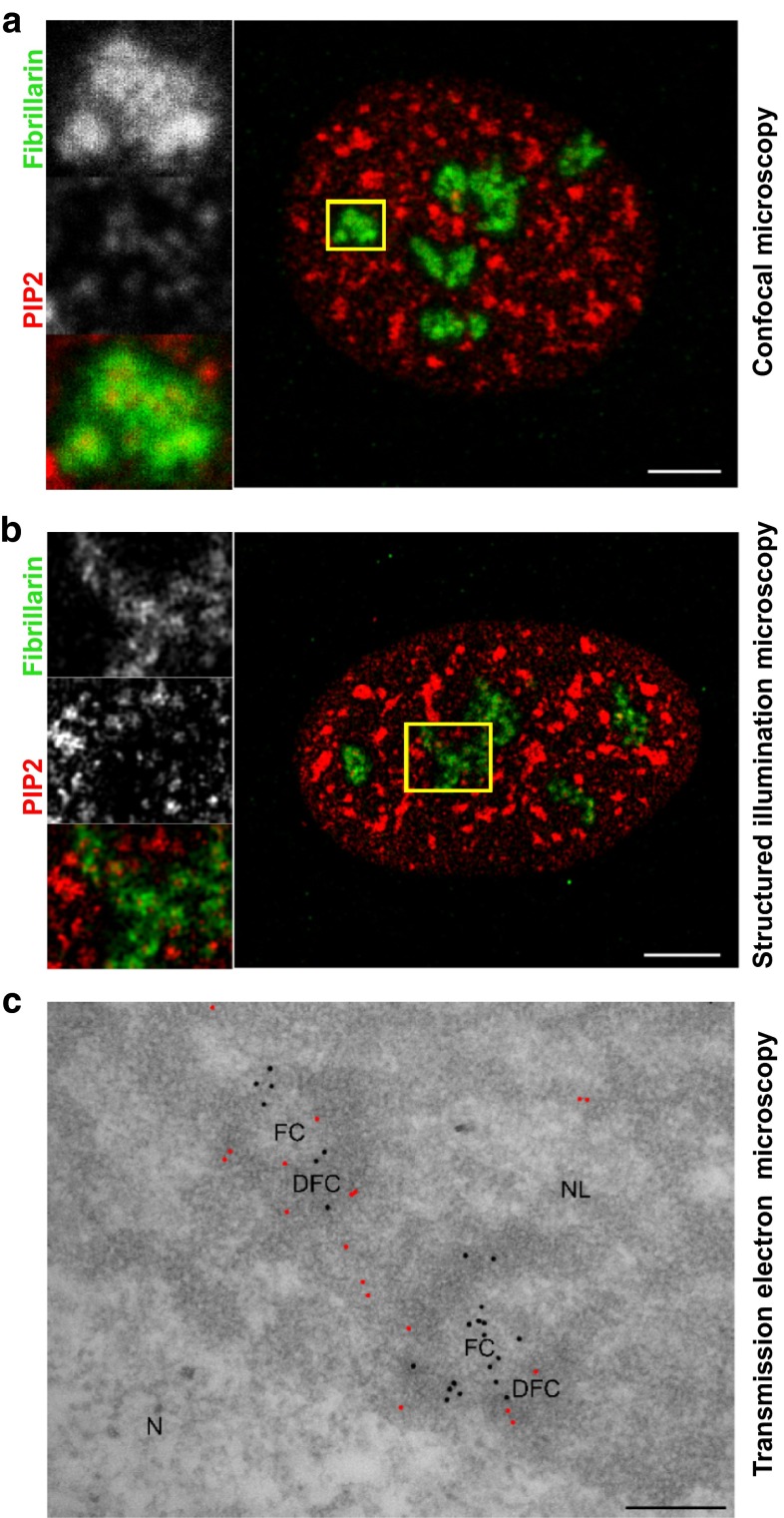
Localization of fibrillarin and PIP2 in the nucleoli in CM, SIM, and EM images with the selected image sections. **a** Fibrillarin and PIP2 detected by confocal fluorescence microscopy. **b** Localization of fibrillarin and PIP2 inspected by SIM. **c** Spatial distribution of PIP2 (*black dots*) and fibrillarin (*red dots*) in EM. FC—fibrillar centers, DFC—dense fibrillar component, N—nucleus, NL—nucleolus.* Scale bars*: 5 µm (A), 5 µm (B) and 100 nm (C)

**Fig. 4:**
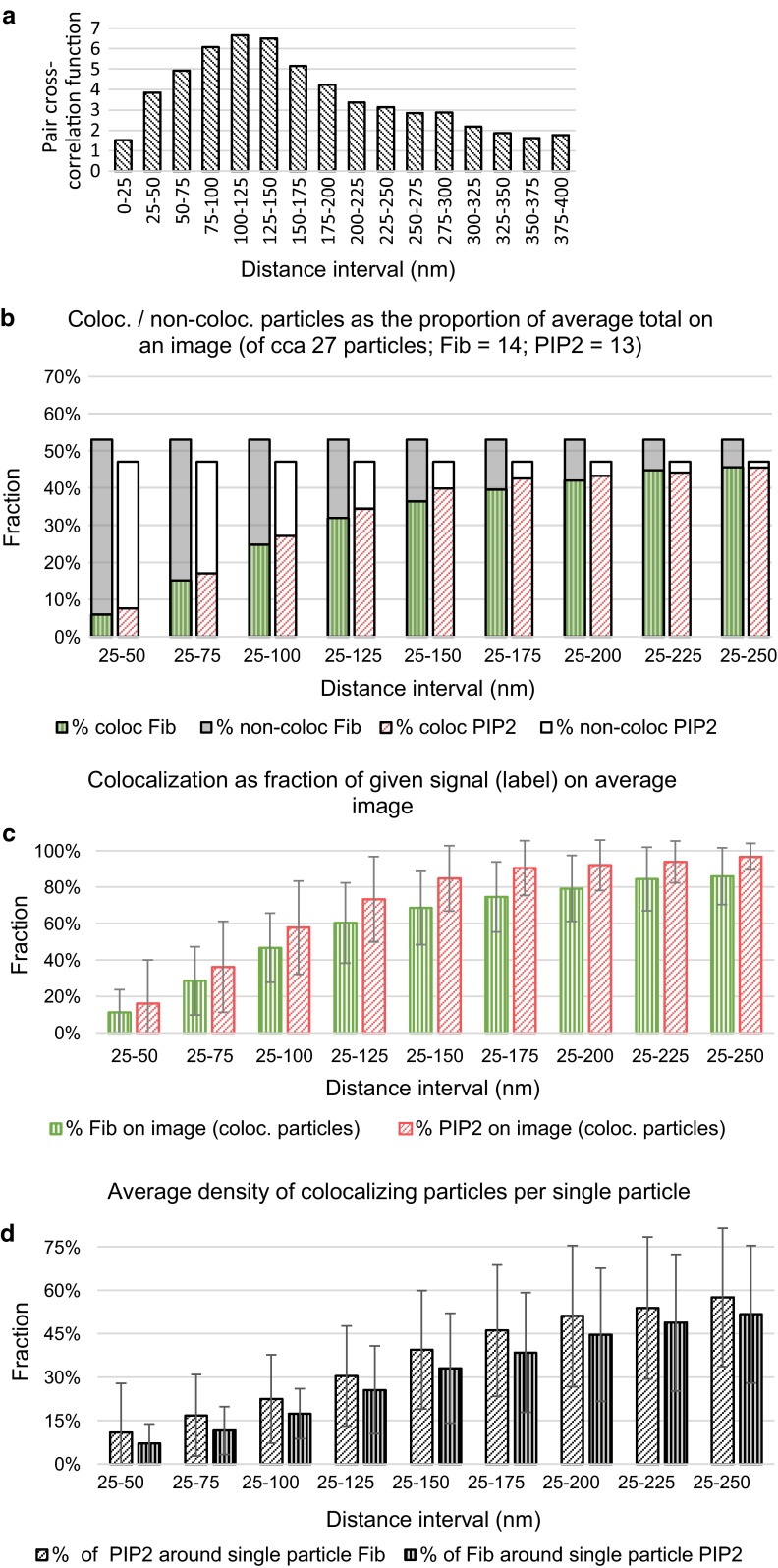
Colocalization data quantified using EM immunolabeling of fibrillarin and PIP2. **a** The pair cross-correlation function compares the empirical frequencies of the pairs of particles on the chosen distance with the theoretical frequency distribution of these pairs. The values higher than value one indicate higher density than theoretically expected for random process. **b** Each pair of the bars represents the relative frequency distribution of the colocalizing and non-colocalizing particles of given label on the given distance range (for detailed visual description of the principle, see Fig. 1). Each shaded or filled area represents the proportion of the particles in the average image. The *dark gray bar* shows the values of the proposed coefficients ${\text{CC}}_{A/(A + B)}^{\text{coloc}}$ (patterned part of the bar) and ${\text{CC}}_{A/(A + B)}^{\text{non-coloc}}$ (*solid part of the bar*). The *white bars* show the values of the coefficients ${\text{CC}}_{B/(A + B)}^{\text{coloc}}$ (*patterned part of the bar*) and ${\text{CC}}_{B/(A + B)}^{\text{non-coloc}}$ (*solid part of the bar*); *A* is fibrillarin and *B* is PIP2. **c** Tendency of the levels of the colocalization coefficients ${\text{CC}}_{2}^{A}$ and ${\text{CC}}_{2}^{B}$ for various distance intervals (mean ± standard deviation). **d** Tendency of the average ratios of colocalizing particles of one type *A* round single particle of another type, represented by the coefficients ${\text{CC}}_{3}^{B/A}$ and ${\text{CC}}_{3}^{A/B}$

The representative images obtained by CM, SIM, and EM are shown in Fig. 3. The EM image data were used to calculate the pair cross-correlation function (PCCF) shown as a graph in Fig. 4a. The levels of PCCF describe, how many times the measured frequency of particles exceeds the theoretical frequency, and they reveal the higher occurrence of the pairs of particles on the distance intervals less than 225 nm.

From Fig. 4b, we can deduce the slightly higher average occurrence of fibrillarin in EM images compared with PIP2. On the other hand, the lower levels of PIP2 particles are associated with a stronger inclination toward colocalization. This is more apparent in Fig. 4c, which illustrates the evolution of the levels of the coefficients ${\text{CC}}_{2}^{A}$ and ${\text{CC}}_{2}^{B}$: The higher proportion of PIP2 is associated with fibrillarin and the lower proportion of fibrillarin is associated with PIP2 (difference between levels >10 %). We can see also the gradual, nearly proportional, increase in the colocalization ratios of both types up to the distance of 125 nm. Afterward, the levels enter the stage of attenuation (upper distance limit >175 nm), and the growth in ratios ceases and transforms into horizontal linear movement. Figure 4d shows the complex insight into the colocalization situation. At the distance interval 25–125 nm, an average particle was associated approximately with 30 % of particles of another type. Combining this information with the levels of the relative colocalization ratios on the same distance interval from Fig. 4b, we can intuitively calculate that an average particle indicating fibrillarin colocalizes approximately with 40 % of colocalizing particles indicating PIP2. Interestingly, an average particle labeling PIP2 colocalizes as well with 40 % of colocalizing particles labeling fibrillarin.

**Fig. 5:**
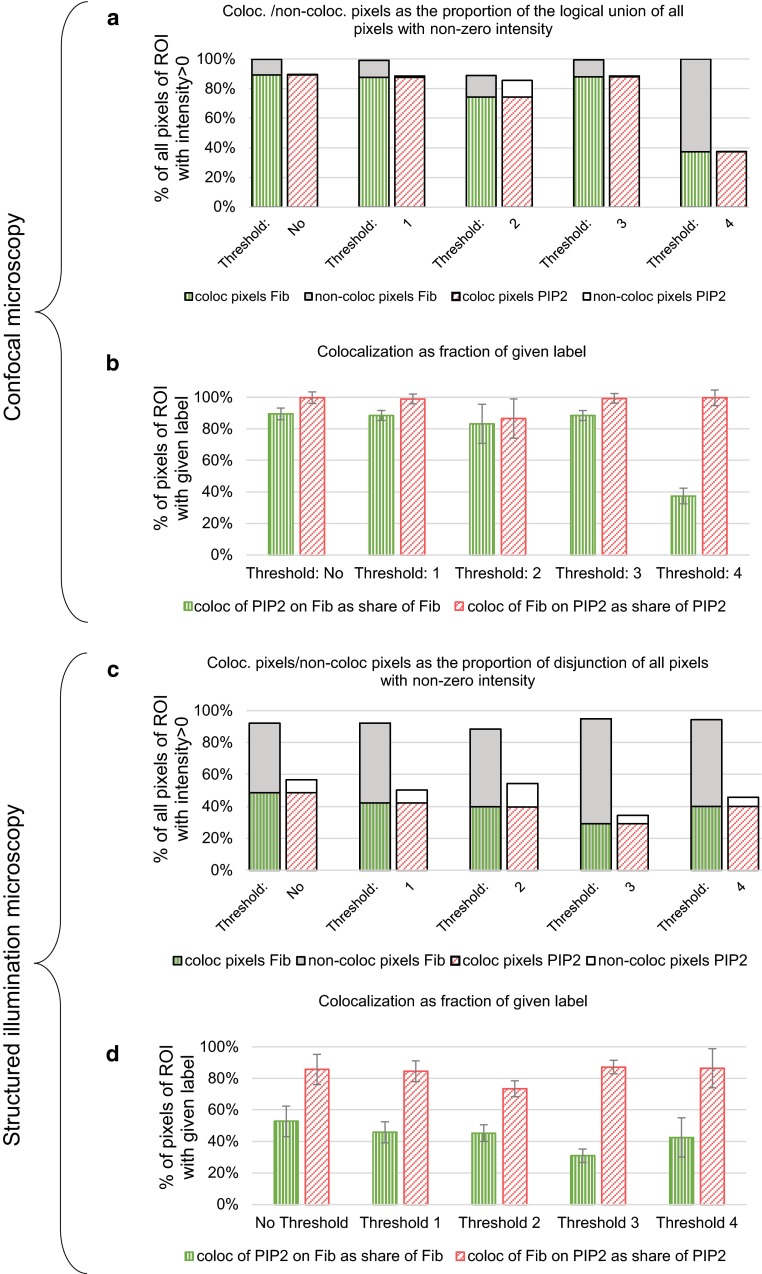
Colocalization between fibrillarin and PIP2 quantified using the data from confocal fluorescence microscopy (**a**, **b**) and super-resolution structured illumination microscopy (**c**, **d**). The *graphs* represent the results of the calculations by the five scenarios of the intensity level adjustments: non-thresholded image (Threshold: No), Costes thresholding based on the image parts of the nucleolar regions of interest (Threshold: *1* and Threshold: *2*) and Costes thresholding based on the whole image (Threshold: *3* and Threshold: *4*). **a**, **c** Each pair of the *bars* represents the average relative frequency distribution of the colocalizing pixels (*patterned part of the bar*) and non-colocalizing pixels (*solid part of the bar*) of the region of interest in the image (for more illustrative description of the principle of quantification, see Fig. 1). **b**, **d** Manders’ overlap coefficients (mean ± standard deviation) calculated from the data in A and C, respectively

The fluorescence data in Fig. 5 show the consistent trend in the colocalization ratios as our proposed colocalization coefficients presented in Fig. 4. Figure 5b, d shows higher abundance of non-colocalized fibrillarin and indicates that PIP2 localization is more dependent on fibrillarin than fibrillarin on PIP2. The values in Fig. 5a, c express the higher spatial enrichment of fibrillarin in the nucleoli compared to PIP2. Remarkably, various thresholding approaches reveal the potential weakness of the accuracy of measuring the colocalization in the analyzed FM images. Also, comparing the data in Fig. 5a, b with the data in Fig. 5c, d, we can conclude that the method of imaging causes by itself the potential bias and may lead to different conclusions.

### Structural comparison of colocalization approaches

From the structural comparison of the colocalization methods used for FM and EM data, we conclude that the accuracy of the EM labeling may be accompanied with the liability of the higher variance in values, which needs to be taken into account. The higher values of standard deviation indicate their dependency on the absolute number of the particles in the images. This in turn depends on the irregularities caused by the physiological state of cells and intracellular compartments as well as variabilities in the accessibility of different areas of a section for antibodies. For statistical tests and higher confidence about the conclusions, this predisposition for uncertainty can be compensated by the changes in sample preparation or statistical outlier detection methods as well as by an increase in the sample size. As the calculations are based on the averaging operation and the averages are likely to be influenced (shifted) by the extreme events (outliers), the robust modifications may eliminate the negative effect of the incorrect localization of the particles. Also, the increased number of the images may help to increase the confidence about the correctness of the estimate of the calculated averages (colocalization ratios). However, equal or roughly equal frequency distributions of the particles between the images, which induce small error bars for the average ratios, are too strict demands underestimating the level of variance driven by the nature of the EM labeling, especially for small image samples.

Also, despite theoretical similarity of the proposed colocalization approach with Manders’ coefficients, it is not fundamentally correct to interpret these approaches in an identical manner. They are based on the principally diverse techniques of sample preparation, detection of molecules, imaging methods, and evaluation approach.

### Differences in sample preparation, imaging methods, and detection of molecules

The procedures of sample preparation for LM and EM are essentially different. In the LM protocol, cells are typically fixed with formaldehyde (FA) and then permeabilized with Triton X-100 to ensure the antibody penetration to the target molecule. FA is a cross-linker forming a methylene bridge (–CH2–) between its single aldehyde group (–CHO) and the nitrogen atom of a peptide linkage or an amino acid side chain. So, proteins are fixed, while lipids and nucleic acids are immobilized through cross-linked protein molecules (Kiernan 2000). There are some data that FA is able to crosslink not only proteins, but DNA as well (Sewell et al. 1984). Hence, an antibody penetrates the perforated cellular membranes and finds its antigen through the cross-linked meshwork of molecules in a cell. The loss of the intracellular material can occur due to the extraction of unfixed molecules through the permeabilized membranes during whole immunolabeling procedure. In the EM protocol, cells are fixed with a mixture of FA plus glutaraldehyde (GA). Then cells are dehydrated and infiltrated with a resin. GA is a much stronger cross-linker than FA, because its two aldehyde groups, separated by a flexible chain of three methylene bridges (HCO–(CH2)3–CHO), react simultaneously with two nitrogen atoms over variable distances (Kiernan 2000; Migneault et al. 2004). It has been reported that GA cross-links only proteins (Sewell et al. 1984). There is a set of data that GA reacts also with phospholipids containing primary amines (phosphatidylserine and phosphatidylethanolamine (Russell and Hopwood 1976) as well as with amino groups of DNA nucleotides (Hopwood 1975). Hence, such strong cross-linking results in a robust immobilization of cell molecules together with more pronounced changes in their conformation. This helps to preserve the cell ultrastructure during following dehydration and resin embedding, but at the same time it complicates the recognition of the target molecule by the antibody. However, some extraction of unlinked molecules can take place during dehydration and infiltration steps. Remarkably, protein extraction takes place even during the freeze substitution (FS) with an organic solvent of high-pressure frozen (HPF) cells (Sobol et al. 2012). It is worthy to note that this effect was reversed by the supplementing of the substitution mixture with 0.5 % glutaraldehyde, which additionally cross-linked the cryoimmobilized protein complexes stabilized by an organic solvent (Sobol et al. 2012). Nonetheless, we should take into consideration that chemical fixation/dehydration and HPF/FS are not identical and trigger different processes in cells. Hence, these methods may result in the retention/extraction of cell molecules in a different manner. That is why in this study we compare the chemically fixed cells further processed for FM or EM. Finally, for EM immunolabeling cells are embedded into acrylic resins, which are better suited for immunocytochemical studies (Roth 1989; Roth et al. 1981; Roth and Taatjes 1998), then a resin-embedded sample is cut into the ultrathin sections (70–90 nm), and an antigen exposed on the section surface is labeled with an antibody. So, we conclude that the procedures for the preparation of LM and EM samples differ principally by the extent of the conformational changes in immobilized molecules, the level of the extraction of unfixed molecules, and the accessibility of a molecule for an antibody.

Additionally, LM allows to visualize molecules in a whole cell volume. Moreover, confocal as well as 3D SIM microscopes allow to scan a cell layer by layer. These features are obviously advantageous, but the resolution is still far away from that of EM: Confocal provides *R*_xy_ = 180–240 nm and *R*_z_ = 460–610 nm; 3D SIM gives *R*_xy_ = 100–130 nm and *R*_z_ = 250–340 nm. EM visualization is done for the labeled section surface only, but it allows the resolution of 0.3–0.5 nm. So, we summarize that LM detects all labeled molecules distributed throughout the cell volume with comparably low resolution. At the same time, EM detects only the molecules labeled on the surface of the ultrathin cell section but with much higher resolution.

Further, a target molecule is recognized by antibody (antibodies) labeled by fluorochrome (for LM) or metallic nanoparticle (EM). In the first case, a fluorochrome is excited with a laser beam of a certain wavelength and LM detects the emission light from a molecule. Due to the resolution limit, we cannot localize precisely a molecule like a single point, but rather as a possible area, where it probably appears. This area is determined by point spread function (PSF) of an optical system. That is why LM visualizes molecules as distribution areas rather than single points and does not allow to distinguish one or more molecules that are located in this area. Hence, measurements of the colocalization in LM cannot capture the subpixel spatial coordinates of the multiple tagged molecules more closely, so the information about the more precise position is optically distorted. In the second case, EM detects a dense gold particle of 6 or 12 nm, which is non-transparent for electron beam. So, EM visualizes nearly directly a molecule of interest.

It should be noticed that there are many factors, which could bias EM as well. Among them, there are various levels of antigen detection, the size of gold particles, the differences in the detection level between antigens *A* and *B*, etc. From the other hand, the modern biology begins to intensively use other super-resolution light microscopy approaches such as stimulated emission depletion (STED) microscopy and stochastic optical reconstruction microscopy (STORM). STED is based on the usage of the stimulated emission depletion beam, which controllably de-excites previously excited fluorophores around the very center of the excitation PSF (Schermelleh et al. 2010). In such a way, the resolution is improved up to *R*_xy_ = 20–100 nm and *R*_z_ = 100 nm (only with z-phase mask, otherwise it is equal to the confocal one). STED surely gives much more precise information about the (co-)localization of the molecules than confocal microscope, but it still operates with PSF of the real position of a molecule. STORM principally differs from SIM and STED because it enables the imaging of single molecules (Fernández-Suárez and Ting 2008). In STORM, the switching of the fluorophores is done stochastically in single-molecule-based manner. In other words, only some molecules are stochastically switched on during each imaging cycle, while the majority of molecules remains dark. The switched-on molecules are imaged and then localized. To reconstruct the whole super-resolution image, we need to repeat this process for many cycles. Final resolution is *R*_xy_ = 20–50 nm and *R*_z_ = 20–30 nm (for 3D-STORM). This pointillistic method allows to determine the position of a single molecule, and hence, it is very promising for an application of our colocalization coefficients, which allow to evaluate the distribution of molecular targets in microscopy methods based on pointed pattern.

### Evaluation method

The ability to distinguish the nearly direct interactions between the molecules from the indirect is much more complicated in FM than in EM images. This becomes apparent especially in the differences of the FM colocalization analysis using Costes thresholding applied to small subcellular or subnuclear structures of interest. The auto-threshold functions may set the cut level too low, which may consequently result in overestimated colocalization (Pompey et al. 2013). Under these circumstances, colocalization results are accompanied by the uncertainty in the correctness of the automatic thresholding. Based on all these critical notions, we conclude that Manders’ coefficients calculated for such small isolated structures within the large images are more advisory and rough estimates than strictly accurate objective ratios. In EM, the histogram of the normalized pair cross-correlation reflects the spatial dependencies and relations of the particles in given distance intervals, so our colocalization approach allows to make a suggestion on the direct interactions and associations between the molecules. Also, FM and EM approaches differ principally due to the dependence of EM coefficients on the chosen distance range, where FM signals are even not resolvable.

Based on the analysis of the antigen patterns, we conclude that the given microscopy technique has a great impact on the final visualization of a cell and an analysis of the imaging data. In terms of the colocalization methods, the presented statistical approach reflects conceptually the spatial codistribution (cooccurrence) of the particles of various types, which is close but essentially different from FM Manders’ pixels overlap of the fluorescent signals from the various channels. Nevertheless, the similar tendency for the values obtained using FM and EM colocalization approaches confirms the accuracy and validity of the scientific conclusions, which are based on the colocalization data.

### Conclusions

Most of the spatial statistical approaches for EM data use the computational motivation from the heavily cited classic publications in theoretical statistics (Diggle 1983; Ripley 1977, 1991, 2005; Stoyan 1990; Stoyan et al. 1987). However, the quantitative analysis of EM data has not progressed as prominently as FM image analysis. This situation bases probably on the fact that EM is considered as an approach requiring expensive instrumentation, training and highly experienced and specialized staff. The EM imaging includes time-consuming and labor-intensive sample preparation and demanding system maintenance. That is why EM is not so widely used, still being very powerful technique with highly valuable output data. But, some influential methods have been developed for quantitative evaluation of the significance of the spatial patterns in EM labeling. On the other hand, any of these statistical EM methods have not addressed the issue of quantification of the colocalization level comparable to FM. For this purpose, our approach extends the current quantitative significance testing in the immunoelectron microscopy images. We establish the principal descriptive colocalization coefficients based on the redefinition of colocalization in the physical terms of the spatial co-distribution of the single points. The colocalization is explained as the systematic cooccurence resulting from the local individual molecular interactions. The presented coefficients connect the theory of complex systems with the EM imaging approach at the analytical and explanatory levels. Moreover, the single-particle colocalization principle can be applied to the other microscopy techniques focused on characterization of the discrete pointed structures (e.g., STED, STORM). The colocalization coefficients in EM and FM share the similar scientific aims, explanatory implications and molecular immunolabeling mechanisms. However, EM and FM techniques vary in sample preparation, imaging procedure, resolution power, quantification methods and robustness. As a consequence, the equality between EM and FM coefficients cannot be clearly expected. Nevertheless, the contradictory colocalization ratios could indicate an incorrect or non-representative biological approach. The important advantage of EM approach compared to FM is the independence on the user’s subjective judgment on the intensity level and thresholding. The EM labeling is based on spatial frequency distribution of distinctive dots in images compared with the optically distorted regions of “higher” intensity in FM.

In this exploratory analysis, we demonstrate the consistency of the proposed original EM approach with the various threshold scenarios in FM colocalization analysis. Based on the recently published data on the involvement of PIP2 in nucleolar processes (Sobol et al. 2013; Yildirim et al. 2013), we investigated the level of the association of PIP2 with fibrillarin in nucleoli. We reveal a higher occurence of fibrillarin compared with PIP2 in nucleoli in both FM and EM images. Remarkably, we show that PIP2 has a higher tendency to colocalize with fibrillarin than vice versa. This confirms our previous findings that PIP2 is required for pre-rRNA processing and it is unlikely present in the nucleolus in an “uninvolved” state.

So we conclude that our proposed approach meets all principal requirements of the modern EM evaluation technique and is applicable to any set of immunolabeling data. However, further complex statistical EM methods derived from this notion will require also the integration of the outlier detection mechanisms and robust modifications, which may lead to more accurate results and less uncertainty. In addition, the combination of the spatial co-occurrence with the spatial clustering implemented as “colocalization of the clustered structures” may bring new insights into the interactions and behavior of the molecular complexes. However, another new definitional schema for explanatory clarity will be necessary for any further development. This future quantitative investigation may bring novel promising results in understanding of the molecular interactions on the ultrastructural level with nanometer resolution.
